# Prolactin response to antipsychotics: An inpatient study

**DOI:** 10.1371/journal.pone.0228648

**Published:** 2020-02-04

**Authors:** Liana Dehelean, Ana-Maria Romosan, Ion Papava, Cristina Ana Bredicean, Victor Dumitrascu, Sorin Ursoniu, Radu-Stefan Romosan

**Affiliations:** 1 Neurosciences Department, Psychiatry Discipline, “Victor Babes” University of Medicine and Pharmacy, Timisoara, Romania; 2 Biochemistry and Pharmacology Department, “Victor Babes” University of Medicine and Pharmacy, Timisoara, Romania; 3 Functional Sciences Department, Public Health Discipline, “Victor Babes” University of Medicine and Pharmacy, Timisoara, Romania; Chiba Daigaku, JAPAN

## Abstract

**Background:**

Antipsychotic medication, stress, gender, and age are factors that influence prolactin levels in patients with psychosis. The aim of the study was to investigate the level of prolactin response to antipsychotic treatment in acute patients, taking into account the total duration of psychosis.

**Methods and findings:**

The study was conducted on 170 acute patients with schizophrenia spectrum disorders and bipolar disorder. Subjects were divided into three subgroups according to the duration of the psychosis (less than 5 years, between 5 and 10 years and more than 10 years of disorder duration). The initial prolactin response under antipsychotic treatment was measured, while the severity of the psychiatric symptoms was assessed with the BPRS (Brief Psychiatric Rating Scale). Hyperprolactinemia was found in 120 (70.6%) patients, amongst which 80 (66.7%) were females and 40 (33.3%) were males. The average increase in prolactinemia was 2.46 times the maximum value in women, and 1.59 times in men. Gender (β = 0.27, p<0.0001), type of antipsychotic medication according to potency of inducing hyperprolactinemia (β = -0.23, p<0.003), and the duration of psychosis over 10 years (β = -0.15, p = 0.04) significantly predicted prolactin levels, when age, diagnosis, antipsychotic category (conventional/atypical/combinations of antipsychotics), and BPRS total scores were controlled for.

**Conclusions and relevance:**

Prolactin levels in patients treated with antipsychotic medication appeared to depend on patients’ gender, on the type of antipsychotic medication according to potency of inducing hyperprolactinemia, and on the duration of the psychosis. An increase in prolactin levels was associated with female gender, while the use of prolactin sparing antipsychotics and a duration of psychosis over 10 years were associated with lower prolactin levels.

## Introduction

Prolactin secretion from the pituitary gland is controlled by the hypothalamus mainly through dopamine (known as prolactin inhibiting factor) and TRH (thyrotropin-releasing hormone), acting as a release factor. Serum prolactin levels may rise in both physiologic (pregnancy, lactation, sleep, stress) and pathologic conditions (brain and systemic diseases). Literature data show that prolactinemia in patients with psychosis is influenced by gender, age [1,2], overt or subclinical hypothyroidism [3], psychotropic medication such as antipsychotics, antidepressants or buspirone [4,5], stress [6–8], and subtype of psychosis [9]. Under antipsychotic treatment, prolactin blood levels may rise up to 10 times the normal values [10]. Prolactin levels over 250 ng/mL are highly indicative for prolactinomas, while drug-induced hyperprolactinemia doesn’t usually exceed 100 ng/mL [11]. The Spanish consensus on the risks and detection of antipsychotic drug-related hyperprolactinemia suggests that prolactin levels over 50 ng/ml or with clinical symptoms require the adaptation of antipsychotic medication (lowering doses, changing the antipsychotic, adding substances known to decrease prolactin levels such as aripiprazole). In case of severe hyperprolactinemia (over 100 ng/ml) the intervention is always recommended, even in the absence of clinical symptomatology, because of the medium and long-term risk of osteoporosis, cardiovascular complications and possible increase in breast or endometrial cancer [12,13].

Dopamine D2 receptor occupancy by antipsychotics predicts the short-term clinical response and hyperprolactinemia, both becoming evident when D2 receptor occupancy exceeds 65%, and 72% respectively [14]. Gruen and collaborators suggest that the magnitude of the prolactin response doesn’t correlate with the clinical response because the maximum level of prolactin is achieved at low antipsychotic doses [15,16]. However, there is also evidence that the degree of the serum prolactin elevation does correlate with clinical response [17]. A study conducted on patients treated with risperidone showed that the baseline prolactin levels may be used to predict the therapeutic response to risperidone [18]. By contrast, Eberhard and collaborators found no significant correlations between prolactin levels and positive or negative symptoms in schizophrenia [19].

Un-medicated women with schizophrenia appear to have lower mean daily prolactin levels than healthy controls [20]. Remitted patients, who relapse, show lower neuroleptic and prolactin serum levels before the relapse episodes than before the stable periods [21]. Thus, prolactin levels may reveal the pathogenic process (the psychosis related hyperdopaminergic state) and, in patients receiving treatment, the antipsychotic biological activity [22].

The response to treatment depends on several factors such as patient and family characteristics (plasma dopamine metabolite homo-vanillic acid) and changes in ventricle volume [23]. Subjects in remission receiving antipsychotic treatment have a normal HPA–hypothalamic–pituitary–adrenal axis function [24], while those experiencing a first episode of psychosis have an activated HPA axis associated with a larger pituitary volume [25]. Conversely, a smaller pituitary volume was found in treated patients with schizophrenia with a duration of illness of at least 5 years, probably due to repeated episodes of HPA axis hyperactivity [25]. However, it is equally possible that smaller pituitary volumes found in patients with schizophrenia may be the consequence of neurodevelopmental impairment [26].

Chronic schizophrenia is associated with low dopamine activity [27]. With subsequent psychotic episodes, a decrease in treatment response, along with illness progression may be acknowledged [28]. The decrease of prolactin response to antipsychotics was seen as the development of a functional tolerance of the dopamine receptors [29]. There is evidence that a number of patients on long term neuroleptic treatment show normal prolactin levels [30]. De Rivera and collaborators found that, in women both young and old, chronic neuroleptic therapy (of at least 5 years) resulted in significantly lower prolactin levels than the acute administration of neuroleptics in similar doses (2–4 weeks). The authors suggested that, in female patients, an adaptation to the neuroleptic blockade of hypothalamic dopaminergic receptors might appear along with the chronic administration of neuroleptic treatment. For male subjects aged 17–45, they proposed that the lack of the chronic effect of neuroleptic treatment might entail that the adaptation only ensues in respect to the estrogen-enhancing mechanism [31].

This study aims to investigate the intensity of prolactin response to antipsychotic treatment in inpatients with psychosis, considering the total duration of the illness and other factors that might have an impact on prolactin serum levels in these patients.

## Materials and methods

### Subjects

One-hundred-and-seventy inpatients with psychosis were included in the study, admitted in the Timisoara Psychiatric Clinic over a period of 6 months. The subjects fulfilled ICD-10 criteria for the following diagnostic categories: schizophrenia (F20), persistent delusional disorder (F22), acute and transient psychotic disorder (F23), schizoaffective disorder (F25), and bipolar disorder–either depressive episode with psychotic symptoms or manic episode with psychotic symptoms (F31). Exclusion criteria were: bipolar disorder—episodes without psychotic symptoms, single manic episode with psychotic symptoms, single depressive episode with psychotic symptoms, recurrent depressive disorder with psychotic symptoms, organic or substance induced psychosis, hepatic or renal failure, pregnancy, lactation, adjunctive medication (such as antihypertensive treatment, gastrointestinal medication, protease inhibitors, estrogens, opiates etc.) that may rise prolactin serum levels [32]. The Scientific Research and Ethics Commission of “Victor Babes” Timisoara University of Medicine and Pharmacy approved the study protocol and the informed consent. This project was conducted in accordance with the Helsinki Declaration. A written informed consent was obtained from each participant in the study.

The authors have undertaken this study as part of their employment, with no funding from any source.

### Procedure

Patients were included in the study randomly, as they were admitted in the psychiatric unit. In order to assess the participants’ level of understanding, the investigators explained the study purpose and methodology in simple terms. Next, the subjects had to respond to questions asked by the investigators related to the content that has just been disclosed. Participants were also asked to give feedback of how their involvement / non-involvement might affect their current situation. Subjects that were considered able to understand the purpose / methodology were presented with the written informed consent. Therefore, capacity testing on an individual basis was carried out by the investigators who observed if each participant understood the question and was able to provide an appropriate answer specific to the situation. All participants underwent a full psychiatric and physical examination. The intensity of the psychiatric symptoms was assessed with the Brief Psychiatric Rating Scale (BPRS) [33]. Psychiatric and medical history were recorded for each patient, in order to obtain data such as: prescribed psychiatric and non-psychiatric medication, co-morbid psychiatric or somatic illnesses, and duration of the psychosis (DP), defined as the time (in years) since first diagnosed psychotic episode. Liver enzymes (aspartate aminotransferase—ASAT, alanine transaminase—ALAT) and serum creatinine were measured to exclude liver and renal failure. The choice of the antipsychotic treatment was left to the psychiatrist supervising the patient.

### Prolactin measurement

At two days after hospital admission, under antipsychotic treatment, serum prolactin levels were measured. The blood samples were collected at 8 a.m., 12 hours after the last dose of antipsychotic. Prolactin levels were determined by the Timisoara Emergency County Hospital using the activated chemo-luminescence method. As recommended by international guidelines, hyperprolactinemia was considered when plasma prolactin levels exceeded 25 ng/ml in women (not pregnant or lactating), and 20 ng/ml in men [34–36].

### Statistical analysis

The collected data were statistically analyzed using SPSS version 20.0 for Windows. The Shapiro-Wilk test revealed that variables were not normally distributed. Therefore, in order to check for differences between groups, we performed non-parametrical tests (the Mann-Whitney U test with the Bonferroni correction, the Kruskal-Wallis test and Dunn-Bonferonni’s post-hoc test). Wilcoxon’s Signed Rank test was applied to examine the potential differences between prolactin levels depending on the duration of psychosis (divided into three subgroups). Potential correlations were checked using Spearman’s correlation coefficients. The χ^2^ test was used for examining differences between categorical data. To test for predictive factors for prolactin serum levels, a multiple regression analysis was performed, for which several values were reported, such as: F (F-test of overall model significance), R^2^ (coefficient of determination), β (unstandardized coefficients), p (level of significance). All continuous variables that were entered in the regression were logarithmically transformed in order to normalize data, while categorical variables were recoded into sets of distinct binary variables. The assumptions needed for running regression analyses were satisfied. The regression was computed taking into account several factors, which were introduced as covariates, so that their influence on the results might be controlled for. Statistical significance was set at p<0.05, and the results were two-tailed.

## Results

### Demographic data

The sample included 170 patients, 59 (34.7%) men and 111 (65.3%) women. Demographic and clinical data are illustrated in Table 1. Hyperprolactinemia was found in 120 (70.6%) patients, of which 80 (66.7%) were females and 40 (33.3%) were males. Of the 80 female patients, 36 (32.4%) were in post-menopause. Significant differences (U = 2194.5, Z = -3.535, p<0.0001) were found between women and men regarding prolactin levels. In women the mean increase in prolactin levels was 2.46 times the normal value, while in men was 1.59 times. Of the female subjects, 75 (67.6%) were still menstruating, while 36 (32.4%) were in post-menopause. Prolactin serum levels did not differ significantly between menstruating and post-menopausal women (p = 0.20). Prolactin levels did not correlate significantly with patients’ age, neither in males (p = 0.11), or females (p = 0.54).

**Table 1:** Demographic and clinical data.

	Men (n = 59): mean, SD	Women (n = 111): mean, SD
Age (years)	39.7 (SD = 12.0)	43.1 (SD = 12.4)
BPRS—total score	54.3 (SD = 13.3)	54.1 (SD = 12.5)
Duration of the psychosis (years)	10.6 (SD = 10.5)	12.3 (SD = 9.5)
Prolactinemia (ng/ml)	31.9 (SD = 23.7)	61.6 (SD = 55.3)

BPRS–Brief Psychiatric Rating Scale, SD–standard deviation

### Clinical data

The distribution of the sample according to the diagnostic subcategory and hyperprolactinemia is shown in Table 2.

**Table 2:** Diagnostic subcategories and hyperprolactinemia.

ICD 10 Diagnostic categories	N = 170, N (%)	Hyperprolactinemia N = 120, N (%)
Schizophrenia (F20)	49 (28.8%)	41 (34.2%)
Persistent delusional disorder (F22)	40 (23.5%)	31 (25.8%)
Acute and transient psychotic disorder (F23)	26 (15.3%)	16 (13.3%)
Schizoaffective disorder (F25)	19 (11.2%)	13 (10.8%)
Bipolar disorder (F31)	36 (21.2%)	19 (15.9%)

N–number of patients

**Fig 1:**
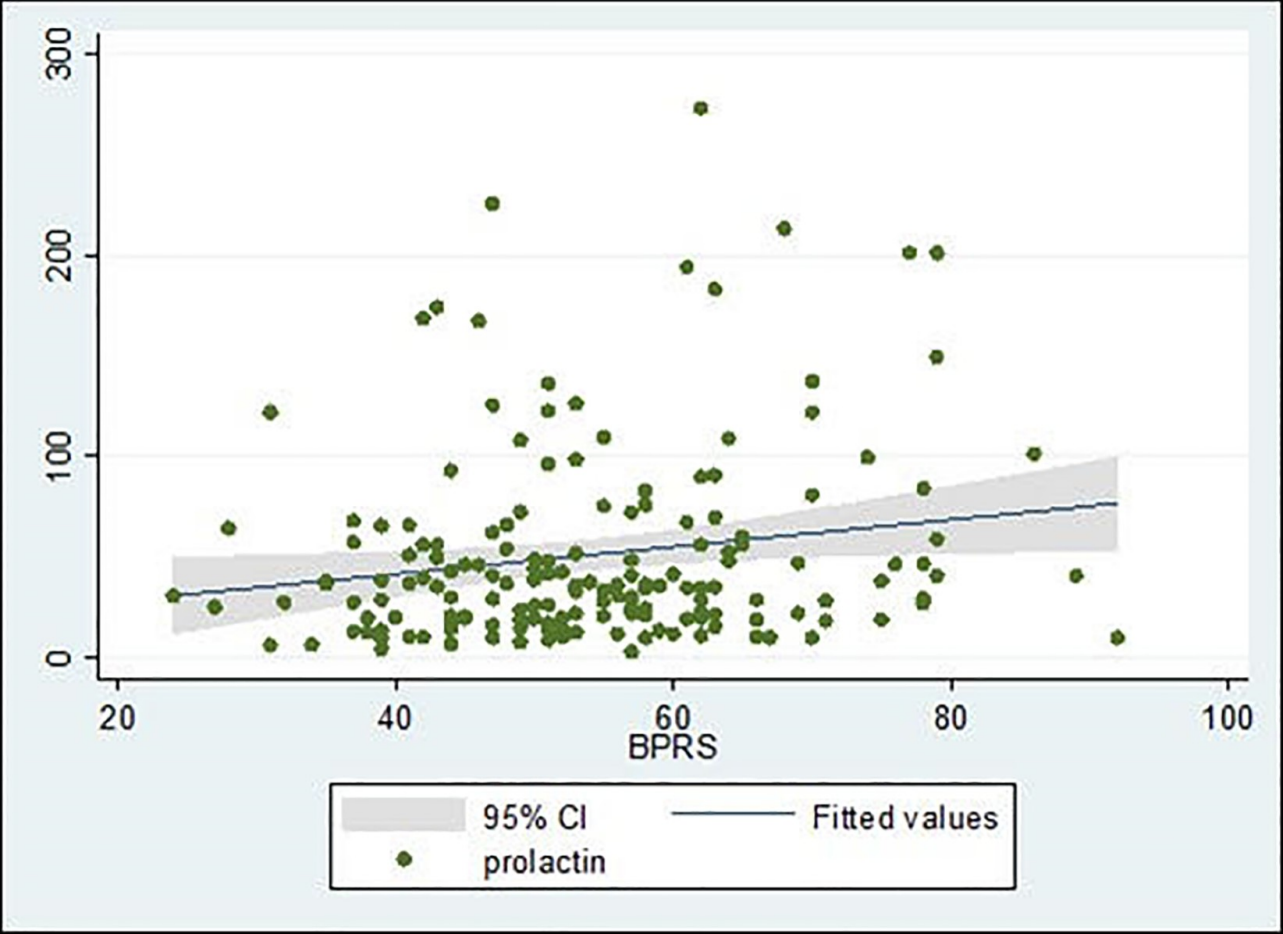
Correlation between BPRS scores and prolactin levels.

The mean BPRS total score in the studied sample was 54.23 (SD = 12.7), with no significant differences between men and women. A statistically significant positive correlation (rs = 0.157, p = 0.042) was found between BPRS scores and prolactin levels (Fig 1).

Sample distribution according to the antipsychotic medication is presented in Table 3. The table also includes the mean dosage for each antipsychotic that was prescribed and its chlorpromazine equivalent [37].

**Table 3:** Sample distribution according to antipsychotic medication and hyperprolactinemia.

Antipsychotic treatment	N (%)	Dosage (mg)	CPZ 100 mg equivalent	Hyperprolactinemia (%)
amisulpride	10 (5.9%)	100	-	9 (90%)
aripiprazole	16 (9.5%)	15	200	3 (18.8%)
clozapine	1 (0.6%)	200	400	1 (100%)
haloperidol	23 (13.5%)	15	750	19 (82.6%)
olanzapine	27 (15.9%)	20	400	23 (85.2%)
quetiapine	25 (14.7%)	800	1066	14 (56%)
risperidone	5 (2.9%)	5	250	4 (80%)
ziprasidone	5 (2.9%)	80	125	4 (80%)
combination of antipsychotics	58 (34.1%)		-	43 (74.1%)

N–number of patients; CPZ–chlorpromazine.

The combinations of antipsychotics are presented in Table 4.

**Table 4:** Combinations of antipsychotics and hyperprolactinemia.

Combinations of antipsychotics (N = 58)	N (%)	Hyperprolactinemia (%)
amisulpride + haloperidol	1 (1.7%)	1 (100%)
haloperidol + levomepromazine	7 (12.1%)	7 (100%)
aripiprazole + haloperidol	3 (5.2%)	2 (66.7%)
aripiprazole + levomepromazine	1 (1.7%)	1 (100%)
olanzapine + amisulpride	1 (1.7%)	1 (100%)
olanzapine + flupenthixol	1 (1.7%)	0
olanzapine + haloperidol	8 (13.8%)	6 (75%)
olanzapine + haloperidol + levomepromazine	4 (6.9%)	4 (100%)
olanzapine + levomepromazine	1 (1.7%)	0
quetiapine + haloperidol	14 (24.2%)	9 (64.3%)
quetiapine + haloperidol + levomepromazine	2 (3.5%)	2 (100%)
quetiapine + levomepromazine	12 (20.7%)	7 (58.3%)
risperidone + haloperidol + levomepromazine	1 (1.7%)	1 (100%)
risperidone + levomepromazine	1 (1.7%)	1 (100%)
ziprasidone + levomepromazine	1 (1.7%)	1 (100%)

N–number of patients.

However, because we had very few patients treated with several antipsychotics (amisulpride, clozapine, risperidone, ziprasidone), patients were classified according to their antipsychotic medication into three categories: receiving exclusively conventional antipsychotics–haloperidol or amisulpride (N = 23, 13.5%), receiving exclusively atypical antipsychotics (N = 89, 52.4%), and receiving a combined antipsychotic treatment (N = 58, 34.1%).

Amongst the 120 subjects presenting hyperprolactinemia, 58 (48.3%) received exclusively atypical antipsychotics, 19 (15.9%) patients received exclusively conventional antipsychotics and 43 (35.8%) patients received a combined antipsychotic treatment. There was no significant association (p = 0.2) between hyperprolactinemia and antipsychotic treatment (atypical/conventional/combinations). Also, there were no significant associations between the type of antipsychotic medication (atypical/conventional/combinations) and patients’ gender (p = 0.15), or diagnosis (p = 0.06). No significant differences were found between patients treated with conventional, atypical and combinations of antipsychotics regarding age (p = 0.52) and DP (p = 0.053).

Antipsychotics were also classified according to potency of inducing hyperprolactinemia in prolactin raising (strongly associated with hyperprolactinemia: haloperidol, amisulpride, risperidone) and prolactin sparing (less associated with hyperprolactinemia: aripiprazole, clozapine, olanzapine, quetiapine, ziprasidone). Patients treated with prolactin raising antipsychotics presented significantly more frequent hyperprolactinemia than those treated with prolactin sparing antipsychotics (p = 0.004). No significant differences were found between patients receiving prolactin sparing and those receiving prolactin raising antipsychotics regarding gender (p = 0.09), age (p = 0.39) and DP (p = 0.97).

**Fig 2:**
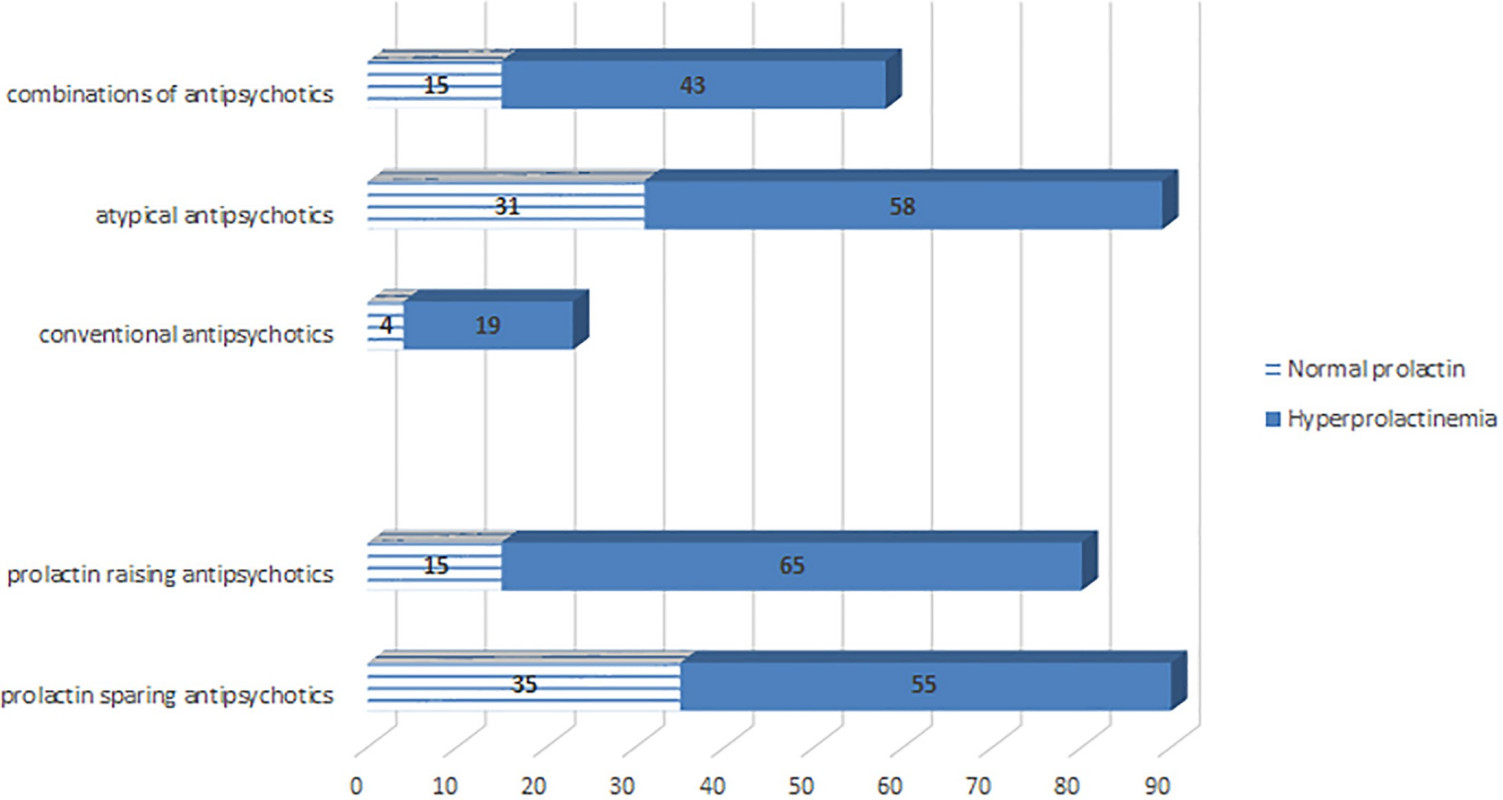
Hyperprolactinemia and prescribed antipsychotic medication.

Sample distribution according to frequency of hyperprolactinemia and antipsychotic medication is illustrated in Fig 2.

The mean (total) duration of the psychosis (DP) for the sample was 11.7 years (SD = 9.9). According to the DP, the subjects were distributed into three subgroups: DP under 5 years (49 patients), DP between 5 and 10 years (31 patients), and DP over 10 years (90 patients). Sample characteristics are presented comparatively for the three DP subgroups in Table 5.

**Table 5:** Sample characteristics according to the duration of the psychosis.

Duration of psychosis	Under 5 years (n = 49)	Between 5 and 10 years (n = 31)	More than 10 years (n = 90)	χ^2^ test or Kruskal-Wallis test
Gender				
men (N = 59)	21 (42.9%)	10 (32.3%)	28 (31.1%)	χ^2^ = 2.03, p = 0.36
women (N = 111)	28 (57.1%)	21 (67.7%)	62 (68.9%)	
Age (years): mean, SD	36.44 (SD = 13.91)	40.06 (SD = 11.94)	45.62 (SD = 10.32)	H = 17.51, p<0.0001***
Diagnosis				
Schizophrenia (N = 49)	7 (14.4%)	10 (32.2%)	32 (35.5%)	χ^2^ = 43.04, p<0.0001***
Acute and transient psychotic disorder (N = 26)	18 (36.7%)	4 (12.9%)	4 (4.4%)	
Persistent delusional disorder (N = 40)	18 (36.7%)	7 (22.6%)	15 (16.7%)	
Schizoaffective disorder (N = 19)	3 (6.1%)	2 (6.5%)	14 (15.6%)	
Bipolar disorder (N = 36)	3 (6.1%)	8 (25.8%)	25 (27.8%)	
BPRS score: mean, SD	57.1 (SD = 12.3)	57.3 (SD = 13.3)	53.7 (SD = 12.5)	H = 1.88, p = 0.39
Antipsychotics				
prolactin raising^a^	27 (55.1%)	10 (32.3%)	43 (47.8%)	χ^2^ = 4.01, p = 0.13
prolactin sparing^b^	22 (44.9%)	21 (67.7%)	47 (52.2%)	
conventional	8 (16.3%)	0	15 (16.7%)	χ^2^ = 8.29, p = 0.08
atypical	21 (42.9%)	21 (67.7%)	47 (52.2%)	
combinations	20 (40.8%)	10 (32.3%)	28 (31.1%)	
Prolactinemia (ng/ml): mean, SD	62.3 (SD = 58.2)	48.6 (SD = 52.3)	46.2 (SD = 41.1)	H = 1.35, p = 0.39
Menstrual cycle (only for female subjects, N = 111)				
women still menstruating (n = 75)	22 (78.6%)	16 (76.2%)	37 (59.7%)	χ^2^ = 4.02, p = 0.13
post-menopausal women (N = 36)	6 (21.4%)	5 (23.8%)	25 (40.3%)	

N–number of patients, SD–standard deviation.

^a^ haloperidol, amisulpride, risperidone

^b^ aripiprazole, clozapine, olanzapine, quetiapine, ziprasidone

***p<0.001.

**Fig 3:**
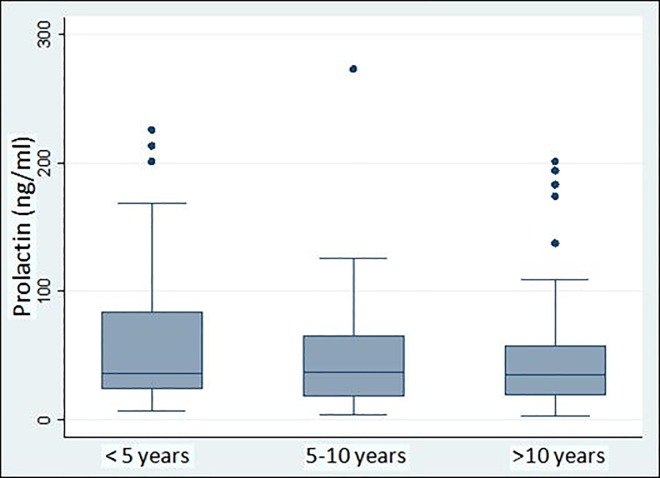
Duration of psychosis and prolactin levels.

Wilcoxon’s Signed Rank test showed that patients with DP over 10 years had significantly lower prolactin levels when compared with those with DP less than 5 years (Z = -2.243, p = 0.025). Prolactin levels were not significantly different between patients with DP ranging from 5 to 10 years and those with DP less than 5 years or over 10 years, as shown in Fig 3.

There were no significant correlations between BPRS scores and DP (p>0.05).

However, when assessing prolactin response to antipsychotics, gender, age, psychopathology and type of medication must be considered. Therefore, a multivariate regression analysis that included all variables that might have an influence on prolactin serum levels was performed: gender, type of antipsychotic medication–prolactin sparing/raising, DP (divided into the aforementioned subgroups), BPRS total scores. Age, diagnosis, antipsychotic category (conventional/atypical/combinations) were also entered in the regression analysis as control variables. The assumptions of homogeneity of variance and linearity of data were met, the residuals were approximately normally distributed, and the collinearity statistics exhibited acceptable results, with variance inflation factor (VIF) scores under 5 for each predictor. The results of the regression showed that the overall model containing all the above mentioned variables was significant, F(13;156) = 3.75, p<0.0001, and explained 24% of the variance. Only gender, type of antipsychotic medication–prolactin sparing/raising, and DP significantly predicted prolactin serum levels, when the other variables where controlled for (Table 6). Female gender was associated with an increase in serum prolactin levels (β = 0.27, p<0.0001), while the use of prolactin sparing antipsychotics (β = -0.23, p<0.003) and a DP over 10 years (β = -0.15, p = 0.04) were associated with lower prolactin levels. The standardized regression coefficients showed that gender had the standardized coefficient with the largest absolute value (beta = 0.35), followed by type of antipsychotic medication—prolactin sparing/raising (beta = 0.31) and the DP over 10 years (beta = 0.21).

**Table 6:** Multiple regression estimates.

Regression model		Unstandardized Coefficients		Standardized Coefficients	t	Level of significance (p)	95% Confidence Interval for B	
		B	SE	Beta			Lower Bound	Upper Bound
1	(Constant)	0.97	0.79		1.22	0.22	-0.59	2.54
	Gender	0.27	0.05	0.35	4.82	<0.0001***	0.16	0.38
	Antipsychotics: sparing/raising	-0.02	0.07	-0.31	-3.05	0.003**	-0.38	-0.08
	BPRS score	0.36	0.26	0.10	1.35	0.17	-0.16	0.88
	Age	0.004	0.30	0.002	0.01	0.98	-0.60	0.61
	Age group: under 50/over 50 years	0.11	0.08	0.13	1.35	0.17	-0.05	0.28
	Conventional antipsychotics	-0.03	0.10	-0.03	-0.37	0.70	-0.24	0.16
	Combinations of antipsychotics	-0.01	0.07	-0.12	-1.32	0.18	-0.24	0.04
	Diagnosis: schizophrenia	0.12	0.08	0.15	1.56	0.10	-0.03	0.28
	Diagnosis: acute and transient psychosis	-0.06	0.10	-0.06	-0.63	0.52	-0.26	0.13
	Diagnosis: schizoaffective disorder	0.13	0.09	0.11	1.34	0.18	-0.06	0.32
	Diagnosis: persistent delusional disorder	0.11	0.08	0.13	1.36	0.17	-0.05	0.29
	DP: between 5–10 years	-0.12	0.08	-0.12	-1.44	0.15	-0.29	0.04
	DP: over 10 years	-0.15	0.07	-0.20	-2.01	0.04*	-0.31	-0.00

DP–duration of psychosis.

*p<0.05

**p<0.01

***p<0.0001.

## Discussions

Conventional or typical antipsychotics are commonly associated with elevated prolactin serum levels, with haloperidol being the most potent drug in terms of its increasing prolactin levels. Amisulpride is another antipsychotic that can cause a significant increase in prolactin serum levels [38,39]. In patients treated with antipsychotics, hyperprolactinemia is the consequence of dopamine D2 receptors blockade in the pituitary gland, which is positioned outside of the blood-brain barrier. The ability to cross the blood-brain barrier is variable for antipsychotics. For example, olanzapine exhibits greater occupancy of brain receptors compared to pituitary receptors [40]. The significant hyperprolactinemia generated by risperidone compared to other atypical antipsychotics might be explained by its lower capacity of passing through the blood-brain barrier and its high affinity for D2 receptors that are present in the pituitary gland [41]. Haloperidol and amisulpride also have lower penetration of the blood-brain barrier [42].

As mentioned above, atypical antipsychotics show variable effects on prolactinemia and can be divided into prolactin raising and prolactin sparing antipsychotics. The majority of atypical antipsychotics, including quetiapine, olanzapine, clozapine, or ziprasidone do not usually generate hyperprolactinemia because of their affinity for both dopamine D2 and serotonin 5-HT2 receptors [41]. Literature shows that the frequency of hyperprolactinemia is highest in patients treated with risperidone (70%–100%). Olanzapine and quetiapine can also be associated with hyperprolactinemia (in 10%–40% of cases), while clozapine usually causes hyperprolactinemia in less than 5% of patients. Nevertheless, the increase of prolactin with these atypical antipsychotics is normally mild [43]. Most of the patients included in the present study showed hyperprolactinemia, regardless of the type of antipsychotic treatment, with the exception of those treated with aripiprazole. This can be explained by aripiprazole’s partial D2 receptor agonism and complete 5-HT2A receptor antagonism [4]. The high percentage of hyperprolactinemia that was found in our patients treated with quetiapine and ziprasidone might be explained by the fact that females were highly prevalent in both the quetiapine group (72% female subjects) and in the ziprasidone group (80% female subjects).

In the present study we found no significant correlations between the prolactin levels and age, although other studies [44,45] have found a higher increase in prolactin levels in younger female patients.

Nevertheless, the assessment of prolactin response to antipsychotics must be conducted taking into account several other factors that might have an influence on prolactin serum levels, such as participants’ gender, age, psychopathology, type of medication, duration of psychosis. The results of our study suggested that prolactin serum levels in patients treated with antipsychotic medication might depend, most of all, on patients’ gender, slightly less on the type of antipsychotic medication–sparing/raising and thirdly, on the duration of the treatment. Female gender was associated with an elevation in serum prolactin levels, while the use of prolactin sparing antipsychotics was associated with lower serum prolactin levels, which appears to be consistent with other studies [46].

As suggested by the results of the regression and Wilcoxon’s Signed Rank test, a DP over 10 years appeared to also significantly influence prolactin serum levels. In subjects with a DP over 10 years, the prolactin serum levels were significantly lower than in patients with DP under 5 years of evolution. Both groups presented similar levels of psychosis induced stress (BRPS scores) and there were no significant differences between patients regarding the antipsychotic treatment (prolactin sparing or raising). The only differences were in the length of the disorder and women to men ratio (1:1 in the group with DP under 5 years, and 2.2:1 in the group with DP over 10 years). In this case, it should be expected that prolactin elevation to be important in the group with DP over 10 years, unless treatment resistance is installed, as the disorder progresses, or treatment adherence is lost. In patients with a DP between 5 and 10 years, the sex ratio was 2:1 in favor of women, and the level of psychosis induced stress was similar to the other two groups, so again, an important prolactin rise should be expected, but these subjects received mainly prolactin sparing antipsychotics. We may infer that the lack of significant prolactin raise in this group might be a consequence of several confounding factors (higher women to men ratio, prolactin sparing antipsychotics and treatment resistance, considering the same levels of disorder induced stress).

In our study, only 3 patients received an adjunctive antidepressant treatment. In this respect, the antidepressant treatment does not constitute a confounding factor for drug-induced hyperprolactinemia. Moreover, the patients did not receive any other medication which may influence prolactin levels.

A limitation of the study is that our results cannot discriminate between high prolactin levels found in antipsychotic free patients with acute psychosis and drug induced hyperprolactinemia, because there was no measurement of prolactin levels before the onset of the current psychotic episode. Another issue that must be taken into consideration is the fact that prolactin levels can also be dependent of menstrual cycles in women, our sample containing 75 (67.6%) females that were still menstruating and 36 (32.4%) female subjects that were in post-menopause. However, prolactin levels were not significantly different between menstruating and post-menopausal women and, although the percentage of post-menopausal women was the greatest in the group with DP over 10 years, the differences between the three DP groups regarding the proportion of menstruating and post-menopausal women were not statistically significant. Therefore, the influence of menstrual cycles on prolactin levels was not accounted for in the regression model, as it appeared to be non-significant in this study. Also, several medical issues related to hyperprolactinemia (such as sexual dysfunctions, risk of osteoporosis etc.) were not assessed in the present study.

The results were interpreted in the absence of thyroid function assessment. However, literature data shows that antipsychotic induced hyperprolactinemia tends to persist despite correction of either subclinical or overt hypothyroidism [47].

## Conclusions

The majority of the patients included in the study showed hyperprolactinemia. Female gender, antipsychotic medication according to the potency of inducing hyperprolactinemia, and the duration of psychosis over 10 years appear to influence prolactin serum levels. We found a progressive decrease in prolactin response after 5 years of evolution, with a significant change after 10 years.
